# Transportation Walking Among US Adults With and Without Transportation Insecurity, 2022 National Health Interview Survey

**DOI:** 10.5888/pcd23.250436

**Published:** 2026-09-17

**Authors:** Graycie Soto, Miriam E. Van Dyke, Jasmine Y. Nakayama, Tiffany J. Chen, Heather M. Devlin, Katherine Irani, Jennifer L. Matjasko, Hatidza Zaganjor, Geoffrey P. Whitfield

**Affiliations:** 1 McKing Consulting Corporation, Atlanta, Georgia; 2 Division of Nutrition, Physical Activity, and Obesity, National Center for Chronic Disease Prevention and Health Promotion, Centers for Disease Control and Prevention, Atlanta, Georgia

## Abstract

**Introduction:**

How Americans get to everyday destinations has implications for health and well-being. Transportation insecurity, or the lack of safe and convenient access to reliable transportation, is a public health problem that can lead to morbidity and mortality through injuries and chronic disease. Walking can be a healthy and low-cost mode of transportation. This study describes transportation walking among US adults with and without transportation insecurity.

**Methods:**

We used 2022 National Health Interview Survey data on 25,889 US adults to estimate the prevalence of transportation walking among adults with and without transportation insecurity across sociodemographic and geographic subgroups. We tested subgroup differences using pairwise comparisons.

**Results:**

In this sample, 5.6% (95% CI, 5.1%–6.2%) of US adults reported transportation insecurity. Walking for transportation was twice as common among adults with transportation insecurity (30.9%; 95% CI, 27.9%–34.1%) than among adults without transportation insecurity (15.3%; 95% CI, 14.5%–16.1%). Among adults with transportation insecurity, the prevalence of transportation walking was higher for adults with the lowest versus the highest levels of educational attainment, adults below versus above their poverty threshold, non-Hispanic Black versus non-Hispanic White adults, younger versus older adults, adults without versus adults with disabilities, and those residing in the West versus the South.

**Conclusion:**

About 1 in 6 US adults walk for transportation, but those with transportation insecurity are twice as likely to do so as those without. Infrastructure and policies that support walking can help improve the health and well-being of Americans and may be especially important for those experiencing transportation insecurity.

**Summary**

**What is already known on this topic?**

Transportation has implications for health and well-being, and walking can be a healthy, low-cost mode of transportation. Transportation insecurity, or the lack of safe and convenient access to reliable transportation, is a public health problem that limits access to important community resources.

**What is added by this report?**

Approximately 5% of US adults reported transportation insecurity. About 16% of adults walked for transportation. Walking for transportation was twice as common among adults with transportation insecurity versus adults without transportation insecurity, a pattern consistent across most sociodemographic groups.

**What are the implications for public health practice?**

Infrastructure and policies that support walking can help improve community health and may be especially important for those experiencing transportation insecurity.

## Introduction

Transportation affects Americans’ health and well-being by providing people with access to food, health care, work, social connection, education, and other everyday needs (1). Transportation insecurity, or the lack of safe and timely access to reliable transportation for everyday activities and needs (2), is a public health problem because it can prevent people from reaping the health benefits of moving freely about their communities (1). Transportation insecurity is more common among certain groups, including adults who experience poverty, have low educational attainment, or identify as non-Hispanic American Indian or Alaska Native (2,3). These groups also face multiple other barriers to healthy living (4,5).

Aerobic physical activity is an important health behavior (6). Walking is the most common form of physical activity in the US (7) and can be a healthy, reliable, and low-cost mode of transportation. Yet *Step it Up! The Surgeon General’s Call to Action to Support Walking and Walkable Communities *notes several barriers to walking for transportation, including not enough time, differences in physical abilities, and a lack of safe built environments that support walking to everyday destinations (8). Built environments that do not adequately support safe walking may be particularly common among several groups susceptible to transportation insecurity (9,10).

In the US, the built environment is often an area of concern because historically, all transportation options have not been prioritized equally (11), resulting in a lack of safe and convenient conditions for using transportation walking to access everyday destinations (12). In many places, the lack of transportation options can limit how Americans get around and where they can go. Because of the history of prioritizing vehicles over other modes of transportation, transportation insecurity in the US is often attributed to a lack of reliable access to a vehicle (13). Those who do not have consistent access to a vehicle may need to walk more frequently on roads designed to move vehicles quickly and efficiently and without adequate pedestrian supports than those who have access to a vehicle (12), which is consistent with findings that transportation walking is often a necessity, not a choice (14). Improving access to safe and convenient walking infrastructure is a public health priority for everyone (8), but the need may be particularly urgent for those experiencing transportation insecurity.

To date, no information is available on the prevalence of transportation walking among people with versus without transportation insecurity. Such information could help guide the prioritization of interventions that support safe walking in communities of greatest need. We compared the prevalence of walking for transportation among US adults with and without transportation insecurity, overall and stratified by sociodemographic and geographic factors.

## Methods

### Design

We used data from the National Health Interview Survey (NHIS), a cross-sectional nationally representative sample of the civilian, noninstitutionalized US adult population that is administered continuously throughout the year (15). The Ethics Review Board at the National Center for Health Statistics (NCHS) approved the survey, and all participants provided verbal consent. All data are deidentified and publicly available (15).

### Sample

We excluded participants who reported they cannot walk at all or climb steps (n = 305) because they were not eligible to answer the assessment of transportation walking. We also excluded participants if they were missing data on transportation insecurity (n = 483), walking for transportation (n = 790), or sociodemographic or geographic characteristics (n = 184). The final analytic sample was 25,889 participants (93.6% of 27,651 total participants) with some sociodemographic differences compared with the excluded participants; for example, the 1,762 participants with missing data were less likely to be non-Hispanic White (50.7% of excluded participants vs 62.9% of included participants) and to have a college degree or higher (26.1% of excluded participants vs 33.0% of included participants).

### Measures

#### Transportation insecurity and transportation walking

For transportation insecurity, participants responded to the question, “In the past 12 months, has a lack of reliable transportation kept you from medical appointments, meetings, work, or from getting things you needed for daily living?” For walking for transportation, participants responded whether they walked in the past 7 days “to travel to and from work, to do errands, or to go from place to place.” We categorized those who answered yes to these questions as having transportation insecurity or walking for transportation, respectively.

#### Sociodemographic and geographic characteristics

Participants self-reported their sociodemographic characteristics: sex (female, male), age (18–24, 25–34, 35–44, 45–64, ≥65 y), race and ethnicity (non-Hispanic American Indian or Alaska Native, single or more than 1 race [AIAN]; non-Hispanic Asian; non-Hispanic Black or African American; Hispanic of any race; non-Hispanic White; non-Hispanic another race or multiple races [another race or multiracial]), and educational attainment (less than high school or General Educational Development [less than high school], high school or General Educational Development, some college or associate’s degree, bachelor’s degree or higher). For disability status, we used the disability status indicator from NHIS designating one’s level of difficulty with 6 functional domains: seeing, hearing, mobility, communication, cognition, and self-care. The indicator classifies participants as having a disability if they responded “a lot of difficulty” or “cannot do at all” to at least 1 domain (15). Participants who responded for the mobility disability status indicator that they cannot walk at all or climb steps were not eligible for the transportation walking assessment in NHIS and were excluded from analyses. Therefore, our disability status variable denotes disabilities among adults with the ability to walk. Geographic residence characteristics included US Census region (16) (Northeast, Midwest, South, West) and urban–rural category using the 2013 NCHS urban–rural classification scheme for counties (large central metropolitan, large fringe metropolitan, medium and small metropolitan, nonmetropolitan) (17). For income, we used an NHIS-provided income–poverty ratio (interpreted as proximity to the federal poverty threshold) that calculates how close a family’s income (or an individual’s income, if the household consists of 1 person) is to the poverty threshold for the family’s size. Ratios less than 1.00 indicate the family lives below poverty, and ratios greater than 1.00 indicate the family lives at or above their poverty threshold (18). The variable we used imputes family income to overcome a high level of missing data, as recommended by NHIS analytic guidance (19). We categorized the income–poverty ratio into 5 categories outlined by Ng et al (20) (<1.00, 1.00–1.99, 2.00–2.99, 3.00–3.99, ≥4.00).

#### Analysis

We estimated the prevalence and 95% CIs of transportation insecurity and, separately, walking for transportation, overall and by sex, age, race and ethnicity, educational attainment, disability status, region, urban–rural category, and proximity to the poverty threshold. Second, we used *t* tests to examine differences between subgroups of each sociodemographic and geographic characteristic in the prevalence of transportation insecurity and, separately, walking for transportation. Third, we described the prevalence of walking for transportation stratified by presence or absence of transportation insecurity, overall and by sociodemographic and geographic characteristics. Lastly, we used *t* tests to examine differences in walking for transportation between subgroups with transportation insecurity (eg, non-Hispanic Black adults with transportation insecurity vs non-Hispanic White adults with transportation insecurity), between subgroups without transportation insecurity (eg, non-Hispanic Black adults without transportation insecurity vs non-Hispanic White adults without transportation insecurity), and across subgroups by transportation insecurity status (eg, non-Hispanic Black adults with transportation insecurity vs non-Hispanic Black adults without transportation insecurity). Significance level was *P* < .05. Where appropriate, we used pairwise testing among subgroups within each characteristic and Bonferroni-corrected pairwise comparisons for multiple comparisons; accordingly, we calculated adjusted significance levels by dividing the standard *P* value threshold by the number of pairwise comparisons for each variable (eg, the adjusted significance level for the 5-level age variable, which has 10 comparisons, is *P* < .005). We conducted statistical analyses with SUDAAN v11.0.1 (RTI International), including the use of weights, to account for NHIS complex survey design and nonresponse. We present only subgroup estimates meeting the NCHS data presentation standards for proportions (19).

## Results

The analytic sample consisted of 14,094 women and 11,795 men (Table 1). In the weighted sample, most participants were aged 45 years or older, identified as non-Hispanic White, had at least some college education or an associate’s degree, did not have a disability, resided in a large metropolitan county, and had income 3 or more times their poverty threshold. The largest share of participants resided in the South.

**Table 1:** Prevalence of US Adults With the Ability to Walk Reporting Transportation Insecurity and Transportation Walking, 2022 National Health Interview Survey^a^

Characteristic	Overall, no. (%)	% Reporting transportation insecurity^b^		% Reporting transportation walking	
		No.	% (95% CI)^c^	No.	% (95% CI)^c^
**Overall**	25,889	1,447	5.6 (5.1–6.2)	4,073	16.1 (15.4–16.9)
**Sex**					
Female^d^	14,094 (51.3)	833	6.0 (5.4–6.6)^e^	2,038	15.0 (14.1–16.0)^e^
Male^e^	11,795 (48.7)	614	5.3 (4.6–5.9)^d^	2,035	17.3 (16.3–18.3)^d^
**Age, y**					
18–24^d^	1,637 (11.6)	145	8.5 (7.1–10.1)^e,f,g,h^	473	27.7 (25.3–30.2)^e,f,g,h^
25–34^e^	3,754 (17.2)	230	6.0 (5.0–7.1)^d,h^	833	20.7 (19.1–22.5)^d,f,g,h^
35–44^f^	4,005 (16.8)	227	5.7 (4.9–6.7)^d,h^	687	16.3 (14.9–17.7)^d,e,g,h^
45–64^g^	8,278 (32.4)	463	5.3 (4.6–6.0)^d^	1,199	13.3 (12.4–14.3)^d,e,f,h^
≥65^h^	8,215 (22.0)	382	4.3 (3.8–4.9)^d,e,f^	881	10.4 (9.5–11.4)^d,e,f,g^
**Race and ethnicity**					
Non-Hispanic American Indian or Alaska Native^d^	346 (1.4)	47	14.1 (9.0–21.4)^e,h^	62	19.5 (14.6–25.7)
Non-Hispanic Asian^e^	1,555 (6.0)	57	3.2 (2.4–4.4)^d,f,g^	334	21.5 (18.9–24.3)^g,h^
Non-Hispanic Black^f^	2,768 (11.3)	255	9.1 (7.9–10.4)^e,h^	500	18.6 (16.5–20.9)^h^
Hispanic or Latino/a^g^	3,642 (17.0)	245	6.8 (5.8–8.0)^e,h^	610	16.5 (15.0–18.1)^e,i^
Non-Hispanic White^h^	17,276 (62.9)	831	4.7 (4.1–5.4)^d,f,g^	2,489	14.8 (13.9–15.7)^e,f,i^
Non-Hispanic another race or multiple race^i^	302 (1.4)	—^j^	—^j^	78	25.9 (20.5–32.0)^g,h^
**Educational attainment**					
Less than high school^d^	2,152 (10.5)	219	9.6 (8.2–11.2)^e,f,g^	352	16.6 (14.8–18.6)^e^
High school or GED^e^	6,494 (26.9)	385	6.1 (5.3–6.9)^d,g^	780	13.2 (12.1–14.4)^d,g^
Some college or associate’s degree^f^	7,312 (29.6)	448	5.9 (5.3–6.7)^d,g^	986	14.8 (13.7–15.9)^g^
Bachelor’s degree or higher^g^	9,931 (33.0)	395	3.7 (3.1–4.5)^d,e,f^	1,955	19.6 (18.4–20.9)^e,f^
**Disability status**					
With disabilities^d^	2,416 (8.4)	346	14.5 (12.6–16.5)^e^	260	12.0 (10.4–13.9)^e^
Without disabilities^e^	23,473 (91.6)	1101	4.8 (4.3–5.4)^d^	3,813	16.5 (15.7–17.3)^d^
**US Census region**					
Northeast^d^	4,225 (17.3)	226	4.9 (4.1–5.9)	977	25.1 (22.7–27.8)^e,f,g^
Midwest^e^	5,705 (20.9)	330	6.4 (4.9–8.3)	886	16.4 (14.7–18.2)^d,f^
South^f^	9,478 (37.9)	522	5.5 (4.9–6.2)	989	10.4 (9.4–11.5)^d,e,g^
West^g^	6,481 (23.8)	369	5.8 (4.9–6.7)	1,221	18.5 (16.9–20.2)^d,f^
**Urban–rural classification**					
Large central metropolitan^d^	7,688 (30.7)	426	5.7 (5.1–6.5)	1,871	23.7 (22.0–25.5)^e,f,g^
Large fringe metropolitan^e^	6,043 (25.2)	267	4.7 (4.1–5.4)	820	14.2 (13.0–15.6)^d,g^
Medium and small metropolitan^f^	8,087 (30.2)	516	6.0 (4.9–7.4)	997	12.9 (11.8–14.2)^d,g^
Nonmetropolitan^g^	4,071 (14.0)	238	6.2 (4.9–7.6)	385	9.7 (8.4–11.3)^d,e,f^
**Income–poverty ratio**					
<1.00^d^	2511 (9.4)	394	15.6 (13.7–17.6)^e,f,g,h^	566	24.0 (21.8–26.3)^e,f,g,h^
1.00–1.99^e^	4,469 (17.6)	392	9.2 (8.2–10.3)^d,f,g,h^	663	16.3 (14.8–17.9)^d,f,g^
2.00–2.99^f^	4,018 (16.1)	198	5.0 (4.2–6.0)^d,e,h^	521	13.2 (11.9–14.6)^d,e,h^
3.00–3.99^g^	3,432 (12.9)	134	3.8 (3.0–4.8)^d,e^	418	12.2 (10.8–13.7)^d,e,h^
≥4.00^h^	11,459 (44.1)	329	2.9 (2.3–3.5)^d,e,f^	1,905	16.6 (15.6–17.7)^d,f,g^

Abbreviation: GED, General Educational Development.

^a^ Percentages and 95% CIs are weighted to account for complex survey design and nonresponse.

^b^ Participants were asked, “In the past 12 months, has a lack of reliable transportation kept you from medical appointments, meetings, work, or from getting things you needed for daily living?” Those answering yes were coded as having transportation insecurity.

^c^ Pairwise comparisons were used to identify differences between subgroups of each sociodemographic and geographic characteristic in prevalence of transportation insecurity and, separately, walking for transportation. Each subgroup is assigned a superscript letter (d, e, f, g, h, i) in the “Characteristic” column. Significant Bonferroni-corrected pairwise comparisons (*P* < .05) between subgroups within each characteristic are indicated with the superscript letters of the significantly different subgroup, next to each respective prevalence estimate (eg, the prevalence of women [“d”] reporting transportation insecurity is significantly different than men [“e”], as indicated by the “e” superscript on the female estimate).

^j^ Estimates not presented due to insufficient sample size based on National Center for Health Statistics guidelines (19).

In this sample, 5.6% (95% CI, 5.1%–6.2%) of adults reported transportation insecurity. The prevalence of transportation insecurity varied considerably across sociodemographic groups. For example, the prevalence of transportation insecurity was higher for people below (<1.00) than for those above their poverty threshold (eg, ranging from 15.6% [95% CI, 13.7%–17.6%] among people below their poverty threshold to 2.9% [95% CI, 2.3%–3.5%] among people with incomes 4.00 or more times their poverty threshold) (Table 1). We found no significant differences in the prevalence of transportation insecurity by geographic characteristics (ie, region and urban–rural classification).

In this sample, 16.1% (95% CI, 15.4%–16.9%) of adults reported walking for transportation. Nonmetropolitan residents had the lowest prevalence of transportation walking (9.7% [95% CI, 8.4%–11.3%]). We found differences across sociodemographic and geographic characteristics. For example, transportation walking was more common among younger adults than older adults: prevalence ranged from 27.7% (95% CI, 25.3%–30.2%) among those aged 18 to 24 years to 10.4% (95% CI, 9.5%–11.4%] among those aged 65 years or older (Table 1).

Adults with transportation insecurity were more likely to walk for transportation (30.9%; 95% CI, 27.9%–34.1%) than adults without transportation insecurity (15.3%; 95% CI, 14.5%–16.1%) (Table 2). This pattern occurred in all examined sociodemographic and geographic subgroups (all *P* values < .01) except among adults with incomes 3 or more times their poverty threshold. We did not test differences among adults identifying as AIAN, non-Hispanic Asian, and another race or multiracial due to insufficient sample size.

**Table 2:** Prevalence of Transportation Walking Among Adults With and Without Transportation Insecurity and Differences Between Subgroups, US, 2022 National Health Interview Survey 2022^a,b^

Characteristic	% Walking for transportation among 1,447 adults with transportation insecurity		% Walking for transportation among 24,442 adults without transportation insecurity	
	No.	% (95% CI)^c^	No.	% (95% CI)^c^
**Overall**	417	30.9 (27.9–34.1)	3,656	15.3 (14.5–16.1)
**Sex**				
Female^d^	247	33.2 (29.4–37.3)	1,791	13.9 (13.0–14.8)^e^
Male^e^	170	28.1 (23.8–32.9)	1,865	16.7 (15.7–17.7)^d^
**Age, y**				
18–24^d^	72	47.9 (39.0–57.0)^f,g,h^	401	25.8 (23.4–28.4)^e,f,g,h^
25–34^e^	95	40.7 (33.5–48.4)^g,h^	738	19.5 (17.8–21.2)^d,f,g,h^
35–44^f^	67	29.3 (23.2–36.3)^d^	620	15.5 (14.1–16.9)^d,e,g,h^
45–64^g^	115	23.4 (19.5–27.8)^d,e^	1,084	12.8 (11.8–13.8)^d,e,f,h^
≥65^h^	68	17.5 (13.0–23.1)^d,e^	813	10.1 (9.2–11.1)^d,e,f,g^
**Race and ethnicity**				
Non-Hispanic American Indian or Alaska Native	—^i^	—^i^	46	15.6 (11.5–20.7)
Non-Hispanic Asian^d^	—^i^	—^i^	311	20.8 (18.2–23.7)^f,g^
Non-Hispanic Black^e^	98	42.6 (35.4–50.2)^g^	402	16.2 (14.1–18.6)
Hispanic or Latino/a^f^	75	32.0 (25.7–39.0)	535	15.4 (13.9–17.0)^d,h^
Non-Hispanic White^g^	199	24.5 (20.8–28.5)^e^	2,290	14.3 (13.4–15.2)^d,h^
Non-Hispanic another race or multiple races^h^	—^i^	—^i^	72	24.5 (19.2–30.7)^f,g^
**Educational attainment**				
Less than high school^d^	77	38.8 (31.6–46.5)^g^	275	14.3 (12.5–16.2)^g^
High school or GED^e^	112	31.0 (25.8–36.7)	668	12.1 (10.9–13.3)^g^
Some college or associate’s degree^f^	119	29.6 (24.8–34.8)	867	13.8 (12.8–15.0)^g^
Bachelor’s degree or higher^g^	109	26.4 (21.7–31.6)^d^	1,846	19.3 (18.1–20.6)^d,e,f^
**Disability status**				
With disabilities^d^	78	25.3 (19.8–31.6)^e^	182	9.8 (8.2–11.6)^e^
Without disabilities^e^	339	32.5 (28.8–36.3)^d^	3,474	15.7 (14.9–16.5)^d^
**US Census region**				
Northeast^d^	75	34.9 (27.7–42.8)	902	24.6 (22.1–27.3)^e,f,g^
Midwest^e^	92	30.9 (24.3–38.4)	794	15.4 (13.8–17.2)^d,f^
South^f^	117	23.6 (19.3–28.5)^g^	872	9.6 (8.7–10.7)^d,e,g^
West^g^	133	39.6 (34.0–45.4)^f^	1,088	17.2 (15.6–18.9)^d,f^
**Urban–rural classification**				
Large central metropolitan^d^	158	36.0 (31.0–41.3)	1,713	23.0 (21.2–24.9)^e,f,g^
Large fringe metropolitan^e^	73	28.8 (22.7–35.8)	747	13.5 (12.3–14.8)^d,g^
Medium and small metropolitan^f^	136	30.5 (25.0–36.7)	861	11.8 (10.7–13.0)^d,g^
Nonmetropolitan^g^	50	24.1 (17.1–32.9)	335	8.8 (7.6–10.2)^d,e,f^
**Income–poverty ratio**				
<1.00^d^	142	39.8 (34.2–45.7)^g,h^	424	21.1 (18.8–23.5)^e,f,g,h^
1.00–1.99^e^	111	33.7 (28.3–39.5)^h^	552	14.6 (13.1–16.1)^d^
2.00–2.99^f^	57	29.4 (22.4–37.5)	464	12.3 (11.1–13.7)^d,h^
3.00–3.99^g^	33	21.5 (13.7–32.0)^d^	385	11.8 (10.4–13.4)^d,h^
≥4.00^h^	74	21.7 (16.8–27.6)^d,e^	1,831	16.5 (15.4–17.5)^d,f,g^

Abbreviation: GED, General Educational Development.

^a^ Percentages and 95% CIs are weighted to account for complex survey design and nonresponse.

^b^ Participants were asked, “In the past 12 months, has a lack of reliable transportation kept you from medical appointments, meetings, work, or from getting things you needed for daily living?” Those answering yes were coded as having transportation insecurity.

^c^ Pairwise comparisons were used to identify differences between subgroups of each sociodemographic and geographic characteristic in prevalence of transportation walking (only where presented), among adults without transportation insecurity and, separately, among adults with transportation insecurity. Each subgroup is assigned a superscript letter (d, e, f, g, h) in the “Characteristic” column. Significant Bonferroni-corrected pairwise comparisons (*P* < .05) between subgroups within each characteristic are indicated with the superscript letters of the significantly different subgroup, next to each respective prevalence estimate (eg, the prevalence of walking for transportation among women [“d”] without transportation insecurity is significantly different than men [“e”] without transportation insecurity, as indicated by the “e” superscript on the female estimate).

^i^ Estimates not presented due to insufficient sample size; based on National Center for Health Statistics guidelines (19).

Among adults with transportation insecurity, we found differences across sociodemographic groups in walking for transportation. For example, walking for transportation was more prevalent among adults aged 18 to 34 years than among adults aged 45 years or older (18–24 y: 47.9% [95% CI, 39.0%–57.0%] and 25–34 y: 40.7% [95% CI, 33.5%–48.4%] vs 45–64 y: 23.4% [95% CI, 19.5%–27.8%] and ≥65 y: 17.5% [95% CI, 13.0%–23.1%]). We also observed higher prevalence of transportation walking among younger adults without transportation insecurity. For example, the prevalence among adults aged 18 to 24 years was significantly higher than the prevalence among all other age groups aged 25 years or older.

Non-Hispanic Black adults with transportation insecurity were more likely to report walking for transportation (42.6%; 95% CI, 35.4%–50.2%) than their non-Hispanic White counterparts (24.5%; 95% CI, 20.8%–28.5%). We observed different patterns by race and ethnicity for those without transportation insecurity.

Among adults with transportation insecurity, people with less than a high school education were more likely to report transportation walking (38.8%; 95% CI, 31.6%–46.5%) compared with those with a bachelor’s degree or higher (26.4%; 95% CI, 21.7%–31.6%). This was notably different than what was observed among adults without transportation insecurity; adults with a bachelor’s degree or higher (19.3%, 18.1%–20.6%) were more likely to walk for transportation than their counterparts with less than a bachelor’s degree, including those with less than a high school education (14.3%; 95% CI, 12.5%–16.2%).

Among adults with transportation insecurity, adults without disabilities were more likely to report walking for transportation (32.5%; 95% CI, 28.8%–36.3%) than adults with disabilities (25.3%; 95% CI, 19.8%–31.6%). Among adults without transportation insecurity, adults without disabilities were more likely to report walking for transportation (15.7%; 95% CI, 14.9%–16.5%) than adults with disabilities (9.8%; 95% CI, 8.2%–11.6%).

Among adults with transportation insecurity, those residing in the West were more likely than those in the South to report walking for transportation (39.6% [95% CI, 34.0%–45.4%] vs 23.6% [95% CI, 19.3%–28.5%]), but we observed no significant differences in transportation walking by urban–rural category. Adults without transportation insecurity living in all other regions of the US were more likely to report walking for transportation than those in the South, and adults without transportation insecurity in large central metropolitan counties were more likely to report walking for transportation (23.0%; 95% CI, 21.2%–24.9%) than in all other urban–rural categories. Those in nonmetropolitan counties were significantly less likely to report walking for transportation (8.8%; 95% CI, 7.6%–10.2%) than those in other categories.

Among adults with transportation insecurity, patterns also varied by income. For example, those below their poverty threshold were more likely to report walking for transportation (39.8%; 95% CI, 34.2%–45.7%) than those with incomes 3.00 to 3.99 times (21.5%; 95% CI, 13.7%–32.0%) and 4.00 or more times their poverty threshold (21.7%; 95% CI, 16.8%–27.6%). Among adults without transportation insecurity, those below their poverty threshold were more likely to walk for transportation than those above their poverty threshold, and those with incomes 4.00 or more times their poverty threshold were more likely to walk for transportation (16.5%; 95% CI, 15.4%–17.5%) than those with incomes 2.00 to 2.99 times (12.3%; 95% CI, 11.1%–13.7%) and 3.00 to 3.99 times their poverty threshold (11.8%; 95% CI, 10.4%–13.4%).

## Discussion

In this nationally representative study, 1 in 20 adults reported transportation insecurity, and 1 in 6 adults reported transportation walking. However, one-third of adults with transportation insecurity reported transportation walking. Transportation walking was generally 1.5 to 2.5 times more common among adults with transportation insecurity than among those without transportation insecurity, overall and among most examined sociodemographic and geographic subgroups. Results from this study reinforce the importance of safe and convenient walking infrastructure to better encourage walking as a mode of transportation and ensure people who do not have reliable access to a vehicle have a safe alternative (1).

Our study found a lower prevalence of transportation insecurity (5.6%) than a previous estimate of 24% among US adults aged 25 years or older (2). The large difference between the 2 estimates could be attributed to how transportation insecurity was assessed. Murphy et al measured many nuances of transportation insecurity using the detailed Transportation Security Index, a validated 16-item instrument that asks participants to report how often they experience symptoms of transportation insecurity (eg, material symptoms such as rescheduling trips or arriving late to destinations, and relational symptoms such as feeling bad or worrying about inconveniencing others) in the past 30 days. In contrast, the transportation insecurity assessment in NHIS asked participants a single yes/no question about their inability to reach specific destinations in the past 12 months. The narrower focus of the NHIS surveillance question could underestimate transportation insecurity.

In all but 2 subgroups, transportation walking was significantly higher among adults with transportation insecurity versus those without. This pattern aligns with recent research showing that many people walk for transportation because they need to, not because they prefer to, especially when they lack reliable transportation options (14). At first glance, this may be a promising finding because many of the transportation–insecure subgroups who reported the highest prevalence of transportation walking (ie, non-Hispanic Black adults, those with less than a high school education, and those below their poverty threshold) have high rates of chronic disease and experience economic challenges (20,21). Given the health benefits of walking and the cost of owning a vehicle (22), these groups may benefit from walking as a health behavior and as a low-cost form of transportation to get to where they need to go. However, a 2024 report from Smart Growth America suggests that several of the groups in our study that were more likely to experience transportation insecurity have high rates of pedestrian injuries and fatalities in the US, including AIAN and Black adults and people living in lower-income areas (12). These high rates may be due to environments that do not support safe walking for these groups, among other factors (23,24). Adults with transportation insecurity who walk for transportation may thus gain health benefits from increased physical activity but may be at greater risk for injury or death while doing so.

No subgroup had a transportation walking prevalence above 50%, regardless of transportation insecurity status. Thus, transportation walking may be an underused source of physical activity, and opportunities exist to increase physical activity levels across the population and to improve health. For example, older adults and adults with disabilities were less likely to report walking for transportation than their counterparts (ie, younger adults and adults without disabilities, respectively), with or without transportation insecurity. Older adults and adults with disabilities have low levels of physical activity during their leisure time (25,26) and have mobility challenges related to the built environment (26,27). Modifying the built environment to facilitate transportation walking may be one way to help these groups achieve the recommended amounts of physical activity overall. Universal design methods, such as curb cuts that remove the need to step up onto the sidewalk and walk signals with audio and visual prompts, can make independent travel easier for people with varying abilities (26). Such improvements can benefit many groups beyond older adults and people with disabilities, such as people pushing strollers or rolling luggage, because they improve safety and ease of movement for everyone (28).

For adults both with and without transportation insecurity, transportation walking tended to be low among those living in the South. Additionally, adults without transportation insecurity living in nonmetropolitan counties had the lowest prevalence of transportation walking compared with all other urban–rural categories. Previous evidence, including some from NHIS, has documented features in the South and rural areas that can make transportation walking less feasible, including fewer perceived supports for walking (29), higher perceived barriers to walking (9), and a lack of destinations (30). This evidence echoes results from the urban planning literature that has found walkability is more than just sidewalks; measures that are strongly related to walking include number of destinations, land-use mix, and connectivity (31,32). Communities in the South and nonmetropolitan counties may consider improving safe options (eg, sidewalks, crosswalks) or zoning policies (eg, allowing mixed use development in downtown cores that may already be walkable) to support more people in using walking for transportation as well as health and well-being.

Our results reinforce the importance of opportunities to improve transportation walking conditions to promote physical activity in the US, especially in areas where transportation insecurity is common. The Centers for Disease Control and Prevention’s Active People, Healthy Nation initiative highlights evidence-based strategies (33) to increase physical activity, which include designing communities that connect activity-friendly and safe routes (eg, sidewalks) to everyday destinations (eg, homes, worksites, schools, grocery stores) and addressing barriers to safe physical activity. By promoting a wide variety of transportation options and infrastructure to support those options (eg, sidewalks, safe street crossings), the varying transportation needs of the US population can be better met, especially in communities that disproportionately experience transportation insecurity and that have a high proportion of residents who walk for transportation (34).

### Limitations

This study has several limitations. First, study data from NHIS relied on self-report and may be subject to recall or social desirability bias. Second, this study did not evaluate whether participants with transportation insecurity had access to other forms of transportation beyond walking (eg, public transit, micromobility, ride sharing), why they reported a lack of reliable transportation (eg, no access to a vehicle, public transit, or a safe place to walk), or whether their environment (eg, safety, traffic, lack of sidewalks) might have influenced their view of walking as a reliable mode of transportation. Third, smaller sample sizes limited the presentation of transportation walking estimates by transportation insecurity status among adults identifying as AIAN, non-Hispanic Asian, and another race or multiracial. Understanding transportation walking in these groups, particularly AIAN adults, who had one of the highest prevalence of transportation insecurity, is important for tailoring solutions to these groups’ contexts and needs. Fourth, the question on reliable transportation asked participants about the past 12 months, while the question on transportation walking asked participants about the past 7 days, which limited our understanding of how the 2 concepts related within the same timeframe. Asking participants about the past 7 days also may not be indicative of their long-term walking patterns. Fifth, people with transportation insecurity and people with a disability were underrepresented among included participants, and this study excluded participants who reported they cannot walk at all or climb steps (1.1% of the initial sample). Exclusions may have biased our results to show lower rates of transportation insecurity: for example, the 1,762 with missing data were less likely to be non-Hispanic White (50.7% of excluded participants vs 62.9% of included participants) and to have a college degree or higher (26.1% of excluded participants vs 33.0% of included participants). Future research could examine infrastructure that supports active modes of transportation that are easier to use for those unable to walk, including universal design methods mentioned above. Finally, NHIS uses a county-based geography measure for urban–rural categorization, which could mask differences by ignoring highly walkable pockets of otherwise rural counties.

### Conclusion

Our study found that about 1 in 6 US adults reported walking for transportation, but those with transportation insecurity were twice as likely as those without transportation insecurity to walk for transportation. Creating safe environments that support walking may allow more people to travel to everyday destinations while being physically active. These improvements may be especially important for people with transportation insecurity, who are more likely to walk for transportation and may face barriers to safe walking. Transportation and land-use practitioners can partner with public health professionals and other sectors to better understand and improve transportation walking conditions for people with and without transportation insecurity.
